# Atherogenic Risk in Shift Versus Non-Shift Workers: Associations with Sociodemographic and Lifestyle Factors

**DOI:** 10.3390/diseases13060188

**Published:** 2025-06-18

**Authors:** Javier Tosoratto, Pedro Juan Tárraga López, Ángel Arturo López-González, Hernán Paublini Oliveira, Carla Busquets-Cortés, José Ignacio Ramirez-Manent

**Affiliations:** 1 Investigation Group ADEMA SALUD, University Institute for Research in Health Sciences (IUNICS), 07010 Palma, Spain; javierlucas.tosoratto@hsll.es (J.T.); h.paublini@eua.edu.es (H.P.O.); c.busquets@eua.edu.es (C.B.-C.); joseignacio.ramirez@ibsalut.es (J.I.R.-M.); 2 Faculty of Medicine, UCLM (University of Castilla La Mancha), 02008 Albacete, Spain; pjtarraga@sescam.jccm.es; 3 SESCAM (Health Service of Castilla La Mancha), 02008 Albacete, Spain; 4 Faculty of Dentistry, University School ADEMA, 07010 Palma, Spain; 5 Institut d’Investigació Sanitària de les Illes Balears (IDISBA), Balearic Islands Health Research Institute Foundation, 07010 Palma, Spain; 6 Balearic Islands Health Service, 07010 Palma, Spain; 7 Faculty of Medicine, University of the Balearic Islands, 07010 Palma, Spain

**Keywords:** shift work, atherogenic risk, physical activity, mediterranean diet, alcohol consumption, smoking

## Abstract

**Introduction.** Atherosclerosis is the histopathological lesion underlying most cardiovascular diseases. Several scales assess the risk of developing atherosclerosis, with the most recognized being atherogenic dyslipidemia (AD) and atherogenic indices (AIs). The aim of this study is to assess the associations between shift work, sociodemographic variables, and lifestyle with atherogenic risk, as determined by atherogenic indices, atherogenic dyslipidemia, and the lipid triad. **Material and Methods.** This is a descriptive, cross-sectional study involving 53,053 workers (28,808 shift workers and 24,245 non-shift workers) from various autonomous communities in Spain and multiple occupational sectors. The association between sociodemographic variables such as age, sex, and socioeconomic status, health habits including tobacco and alcohol consumption, physical activity (PA), adherence to the Mediterranean diet (MD), and shift work with the presence of AD and high values of three AIs (Cholesterol/HDL-c, LDL-c/HDL-c, and Triglycerides/HDL-c) were assessed. **Results.** All variables analyzed were associated with AD and AIs values. Among the variables, the strongest associations were observed for physical activity, with odds ratios (ORs) ranging from 7.70 (95% CI: 6.86–8.55) for high LDL-c/HDL-c to 14.10 (95% CI: 9.05–14.16) for AD; adherence to the Mediterranean diet, with ORs ranging from 1.98 (95% CI: 1.60–2.37) for high LDL-c/HDL-c to 5.89 (95% CI: 4.92–6.86) for AD; and age, with ORs of 2.11 (95% CI: 1.84–2.38) for high Triglycerides/HDL-c and 4.66 (95% CI: 4.04–5.28) for high Total Cholesterol/HDL-c. **Conclusions.** The profile of a worker with the highest atherogenic risk in our study is a male, older in age, with low socioeconomic status, a smoker, a habitual alcohol consumer, physically inactive, with low adherence to the Mediterranean diet, and engaged in shift work.

## 1. Introduction

Shift work, defined as employment conducted outside the conventional working hours of 7 a.m. to 6 p.m. [1], has gained increasing relevance in contemporary societies due to the rising demand for continuous services in sectors such as healthcare [2], industry [3], security [4], and transportation [5]. Shift work is essential for economic and social functioning, but it poses significant health risks for workers, which has attracted the attention of the scientific and healthcare communities [6].

According to the International Labour Organization (ILO), more than 20% of the global working population is engaged in some form of shift work, and in certain regions and specific sectors this proportion may exceed 40% [7]. In industrialized countries, the prevalence of night work is particularly high, affecting an estimated 15% to 25% of the workforce, and in the United States, it accounts for approximately 20% [8].

The high prevalence of shift work is not only noteworthy in itself, but also because it disrupts the circadian rhythm, which is essential for regulating key physiological functions such as sleep, metabolism, and hormonal secretion [9]. The high prevalence of shift work extends beyond its frequency to encompass an increased risk of health problems, including sleep disorders, psychological conditions, and chronic diseases—particularly cardiovascular and metabolic disorders—highlighting its detrimental impact on health [10].

The clinical implications of shift work are profound and multifaceted. One of the most well-documented effects is chronic sleep deprivation [11], which negatively impacts mental health and cognitive performance, especially in women [12]. Additionally, circadian disruption increases susceptibility to metabolic disorders, including obesity, type 2 diabetes, and dyslipidemia. Hormonal disturbances, such as elevated cortisol levels and insulin resistance, are among the key underlying mechanisms explaining these associations, with differences between the sexes [13].

Moreover, shift work has been linked to a heightened incidence of cardiovascular diseases [14], such as hypertension [15], coronary artery disease [16], and heart failure [17]. Shift work is associated with up to a 40% increased risk of cardiovascular events compared to daytime work. This elevated risk has been linked to unhealthy lifestyle factors, chronic inflammation, and endothelial dysfunction, as reported in various epidemiological studies [18].

Atherosclerosis, the underlying process in most cardiovascular diseases, is associated with the accumulation of atherogenic lipoproteins and chronic vascular endothelial inflammation [19]. Atherogenic indices, such as the total cholesterol/HDL ratio and the triglycerides/HDL ratio, have been widely used as markers of cardiovascular risk across diverse populations [20,21,22]. In shift workers, these indices may be altered, reflecting an adverse lipid profile [23].

Atherogenic dyslipidemia, characterized by elevated triglycerides, reduced HDL cholesterol, and a predominance of small, dense low-density lipoproteins (small dense LDLs), is a common phenotype in shift workers [24]. This phenotype, along with elevated atherogenic indices, not only increases the risk of atherosclerosis but also predisposes individuals to severe clinical events such as myocardial infarction [25] and stroke [26]. Chronic exposure to shift work-related stressors, including sleep deprivation and psychosocial stress, plays a critical role in the development of these lipid alterations [27].

The interplay between cardiometabolic and atherogenic risks in shift workers underscores the need for an integrated approach to their assessment and management. The concomitant presence of metabolic syndrome [28], hypertriglyceridemic waist [29], and atherogenic dyslipidemia not only amplifies overall cardiovascular risk but also complicates the prevention and treatment of these conditions. Furthermore, occupational and social factors specific to shift workers, such as limited access to health promotion programs and the insufficient adaptation of healthcare systems to their needs, exacerbate the situation.

The objective of this study is to evaluate the associations between shift work, sociodemographic variables (e.g., age, sex, and socioeconomic status), and health-related habits (e.g., tobacco or alcohol consumption, physical activity, and adherence to the Mediterranean diet) with atherogenic risk, as determined by atherogenic indices, atherogenic dyslipidemia, and the lipid triad.

## 2. Materials and Methods

### 2.1. Participants

**Figure 1:**
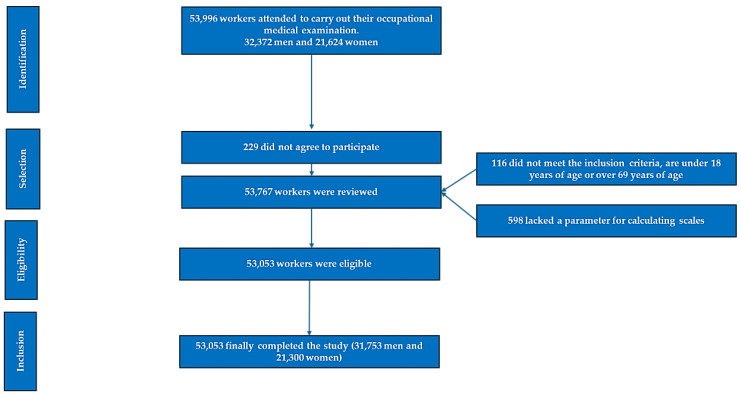
A flowchart depicting participant selection after applying the inclusion criteria.

This observational, cross-sectional, and descriptive study involved a cohort of 53,053 workers representing nearly all sectors of employment across various regions of Spain. The sample consisted of 31,753 men (17,527 of whom were shift workers) and 21,300 women (11,281 of whom worked shifts). We included data from workers employed in 74 companies across various sectors (industry, healthcare, security, hospitality, and services) in several autonomous communities in Spain. Participants were selected based on their inclusion in the annual routine medical assessments conducted by the collaborating companies, after applying the inclusion criteria (Figure 1). All data were collected during these routine medical check-ups carried out between January 2019 and June 2020.

Sociodemographic variables included sex, age (grouped as 18–29, 30–39, 40–49, 50–59, and 60–69 years), occupational social class (I–III, based on the Spanish National Classification of Occupations), and educational level (primary, secondary, and university). Lifestyle variables included tobacco and alcohol consumption (yes/no), adherence to the Mediterranean diet (assessed using the MEDAS index, categorized as low or high), and physical activity (active/inactive). Shift work was defined as any work schedule outside of the standard daytime hours.

Primary outcomes were the presence of atherogenic dyslipidemia (AD) and elevated values in the following three lipid-based atherogenic indices: total cholesterol/HDL-c (Castelli index), LDL-c/HDL-c (Kannel index), and triglycerides/HDL-c ratio. These were dichotomized according to established cut-off points from the literature.

Descriptive analyses and bivariate comparisons were performed. Multivariate logistic regression models were then used to examine associations between independent variables and each outcome. Odds ratios (ORs) and 95% confidence intervals (95% CIs) were reported. Model fit was evaluated using Nagelkerke’s R^2^. To reduce the risk of type I error due to multiple comparisons, exploratory analyses with Bonferroni correction were conducted (see Supplementary Table S2).

The inclusion criteria are as follows:

- Age range: 18–69 years.
- Active employment under a contractual agreement with one of the participating companies.
- Informed consent to participate in the study.
- Approval of the use of their medical data for epidemiological research.

### 2.2. Variable Assessment

Information was collected by trained healthcare professionals affiliated with the occupational health services of the participating companies. To minimize interobserver variability, all procedures were standardized in advance. Anamnesis served as one of the primary data collection methods, capturing information on sociodemographic characteristics (age, sex, socioeconomic status, and educational attainment) and health-related behaviors (smoking, alcohol consumption, adherence to the Mediterranean diet, and physical activity).

The clinical and anthropometric measurements are as follows:

- Data on height, weight, waist circumference, and blood pressure (systolic and diastolic) were collected.
- The analytical measurements are as follows:
- Blood glucose and lipid profile assessments were performed.
- To minimize bias, standardized protocols for the measurement and assessment of all variables were rigorously applied.

#### 2.2.1. Anthropometric Measurements

Participants’ height and weight were measured while standing upright, wearing minimal clothing, and with arms at their sides, ensuring alignment of the head and chest. Measurements were taken in kilograms and millimeters using SECA-standard equipment following ISAK anthropometric guidelines [30].

Waist circumference was determined with a SECA measuring tape, positioned horizontally at the midpoint between the last rib and the iliac crest while participants stood in a relaxed posture. Hip circumference was recorded at the widest part of the buttocks using the same method.

#### 2.2.2. Clinical Measurements

Blood pressure was assessed using an OMROM-M3 device after participants had rested for at least 10 min in a seated position. Multiple cuff sizes ensured a proper fit. Three consecutive readings were taken at one-minute intervals, with the average of these readings used as the final value. Hypertension was defined as systolic pressure ≥ 140 mmHg, diastolic pressure ≥ 90 mmHg, or ongoing antihypertensive treatment.

#### 2.2.3. Analytical Measurements

Venous blood samples were obtained following a 12 h fasting period. Samples were refrigerated for 48–72 h before analysis in accredited laboratories, adhering to standardized protocols. Blood glucose, triglycerides, and total cholesterol were quantified using enzymatic methods, while HDL cholesterol levels were determined through precipitation techniques. LDL cholesterol was calculated using the Friedewald formula [31] unless triglycerides exceeded 400 mg/dL, in which case direct measurement was used. Analytical values were expressed in mg/dL, with dyslipidemia defined as lipid levels exceeding laboratory reference thresholds or the use of lipid-lowering medication.

#### 2.2.4. Atherogenic Risk Assessment

Atherogenic dyslipidemia was defined by triglyceride levels ≥ 150 mg/dL combined with HDL cholesterol < 40 mg/dL in men or <50 mg/dL in women, with LDL cholesterol < 160 mg/dL [32]. The presence of all three markers (elevated triglycerides, low HDL, and high LDL > 160 mg/dL) was classified as the lipid triad [33].

The following three atherogenic indices were calculated:

1. Total cholesterol/HDL cholesterol (Castelli index).
2. LDL cholesterol/HDL cholesterol (Kannel index).
3. Triglycerides/HDL cholesterol.

Cutoff values for these indices are as follows [34]:

- Castelli index: Low (<5% for men); moderate (4.5–7% for women, 5–9% for men); high (>7% for women, >9% for men).
- Kannel index: High risk > 3%.
- Triglyceride/HDL ratio: High risk > 3%.
- Additional sociodemographic variables are as follows:
- Sex: Classified as male or female.
- Age: Calculated as the difference between the date of medical examination and date of birth.
- Education level: Categorized into primary, secondary, and university-level studies.
- Socioeconomic status: Determined using the Spanish Society of Epidemiology guidelines based on the following occupational classifications in the 2011 National Classification of Occupations (CNO-11) [35]:
  ○ Class I: University-educated professionals, managers, athletes, and artists.
  ○ Class II: Intermediate professionals, skilled self-employed workers.
  ○ Class III: Workers with limited qualifications.
- Smoking status: Defined as smoking any tobacco product within the past 30 days or abstaining from smoking for less than one year.
- Adherence to the Mediterranean diet: Evaluated via a 14-item questionnaire, with scores ≥9 indicating high adherence [36].
- Physical activity: Assessed using the International Physical Activity Questionnaire (IPAQ), which measures activity levels over the previous week [37].
- Alcohol consumption: Quantified in standard drinking units (SDUs), with one SDU equaling 10 g of pure alcohol (equivalent to 100 mL wine, 100 mL champagne, 200 mL beer, or 25 mL spirits). Excessive consumption was defined as >35 SDUs/week for men and >20 SDUs/week for women [38].

### 2.3. Statistical Analysis

Descriptive statistics were used to summarize categorical variables as frequencies and distributions, while the normality of continuous variables was assessed via the Kolmogorov–Smirnov test. Means and standard deviations were calculated for quantitative variables. Bivariate analyses included Student’s *t*-test for mean comparisons and Chi-square tests for proportions. Multinomial logistic regression was employed to examine variables associated with elevated atherogenic risk, with goodness-of-fit evaluated using the Hosmer–Lemeshow test. Stratified analyses to control for confounding factors did not identify significant effects. Statistical analyses were conducted using SPSS (version 29.0), with significance set at *p* < 0.05.

## 3. Results

Table 1 summarizes the anthropometric, clinical, analytical, and sociodemographic characteristics of participants, stratified by sex and shift work status.

Shift workers exhibited less favorable anthropometric, clinical, and analytical profiles compared to non-shift workers, with men consistently showing worse outcomes except for HDL cholesterol. The differences between shift and non-shift workers were statistically significant for all parameters except height.

The majority of participants were aged 30–49 years and belonged to the lowest socioeconomic class (Class III), with elementary-level education. Smoking prevalence was higher among male shift workers and female non-shift workers. Higher adherence to the Mediterranean diet and greater physical activity were observed in non-shift workers of both sexes. Alcohol consumption was significantly elevated in shift workers.

Table 2 and Table 3 present the mean values and prevalence of high-risk levels for atherogenic risk scales (atherogenic indices and atherogenic dyslipidemia) based on sociodemographic variables (age and socioeconomic status), health habits (smoking, alcohol consumption, physical activity, and adherence to the Mediterranean diet), and shift work in both sexes. The data indicate that both mean values and the prevalence of high atherogenic risk increase with advancing age, lower socioeconomic status, unhealthy habits (smoking, habitual alcohol consumption, physical inactivity, or low adherence to the Mediterranean diet), and among shift workers. In all cases, men exhibit more unfavorable values compared to women. The observed differences are statistically significant (*p* < 0.001).

The results of the multinomial logistic regression analysis (Table 4) reveal that all independent variables included in the model (sex, age, socioeconomic status, smoking, alcohol consumption, physical activity, adherence to the Mediterranean diet, and shift work) are associated with an increased risk of atherogenic dyslipidemia and high levels in the three atherogenic indices. Among these variables, those with the highest odds ratios are physical activity, adherence to the Mediterranean diet, and age.

The sample consisted of 56.7% men and 43.3% women, with a mean age of 43.1 ± 10.2 years. The overall prevalence of atherogenic dyslipidemia was 11.2%, while the proportions of individuals with elevated lipid indices ranged from 14.5% (high LDL-c/HDL-c) to 21.8% (high TG/HDL-c).

Multivariate logistic regression analyses revealed that all key sociodemographic and lifestyle factors were significantly associated with increased atherogenic risk. Male sex, older age, lower social class, lower educational level, smoking, alcohol consumption, low adherence to the Mediterranean diet, physical inactivity, and shift work were all independently associated with higher odds of presenting atherogenic dyslipidemia or elevated lipid indices.

Model goodness-of-fit was acceptable, with Nagelkerke R^2^ values of 0.242 for atherogenic dyslipidemia, 0.228 for high TC/HDL-c, 0.217 for high LDL-c/HDL-c, and 0.236 for high TG/HDL-c. After applying Bonferroni correction in exploratory analyses, all key associations remained statistically significant (adjusted *p* < 0.009), reinforcing the robustness of the findings (see Supplementary Table S2).

## 4. Discussion

Atherogenic indices provide an accurate assessment of cardiovascular risk by considering the balance between harmful (LDLs, triglycerides) and protective (HDLs) lipoproteins. In our study, these indices are linked to demographic and lifestyle factors that influence lipid metabolism. We analyzed how variables such as age, sex, socioeconomic status, smoking, alcohol consumption, physical activity, adherence to the Mediterranean diet, and shift work are associated with atherogenic risk, offering a comprehensive perspective on their impact on cardiovascular health.

In our study, age emerged as one of the main variables influencing atherogenic indices. These findings are consistent with previous studies [39]. The increase in atherogenic indices with age was observed in both men and women, with higher values among shift workers. Age-related physiological hormonal and metabolic changes lead to elevated total cholesterol and LDL levels, along with decreased HDL levels, all of which contribute to increased cardiovascular risk [40]. In men, aging is associated with an increase in LDLs and a decrease in HDLs, influenced by the decline in testosterone levels [41]. In women, estrogens exert a protective effect on the lipid profile, promoting higher HDL levels before menopause [42]. However, after menopause, this protection diminishes, leading to a lipid profile more similar to that of men, characterized by increased LDL and decreased HDL levels [43]. Longitudinal studies, such as those conducted by the American Heart Association, have shown that cardiovascular risk is lower in women up to the age of 50, becoming comparable to that of men after menopause [44]. Moreover, age is linked to an increased risk of dyslipidemia, obesity [45], and hypertension [46], all of which negatively impact atherogenic indices. Therefore, aging is a key determinant in the assessment of cardiovascular risk and in the interpretation of lipid profiles.

In our research, socioeconomic status (SES) was represented by two variables: social class and educational level. In both cases, a significant association was observed with the different atherogenic indices and the presence of dyslipidemia. The results show a progressive increase in both the percentage of affected individuals and the odds ratios as educational level decreases and social class descends. This trend was consistent in both men and women, although it was more pronounced among shift workers compared to non-shift workers. Several studies have demonstrated that lower SES is associated with more unfavorable lipid profiles, characterized by higher levels of LDL and triglycerides and lower HDL levels [47]. This relationship is explained by behavioral factors (such as poorer diets, higher smoking rates, and physical inactivity) and environmental factors (limited access to healthy foods, preventive services, and safe spaces for physical activity) [48,49]. According to the PREDIMED-plus study, individuals with lower SES have a higher likelihood of developing metabolic syndrome [50]. Moreover, dyslipidemia is more prevalent in these groups, as reflected in higher atherogenic indices. These disparities are further exacerbated by adverse working conditions and less healthy lifestyles, increasing their cardiovascular risk [51]. Therefore, implementing public health policies focused on equity is essential to reduce metabolic disparities and improve lipid profiles in socioeconomically vulnerable populations [52].

In our findings, smoking was found to be negatively associated with lipid profile parameters. Although shift workers showed a higher prevalence of atherogenic dyslipidemia and elevated atherogenic index values, the odds ratio was similar between shift and non-shift workers. This may be due to the comparable number of smokers in both groups, suggesting that shift work may be linked to other unhealthy behaviors which, in combination with smoking, adversely affect lipid profiles. Previous studies have confirmed that smoking increases triglyceride levels and reduces HDL, thereby increasing the risk of dyslipidemia and atherosclerosis [53]. Smokers tend to exhibit higher levels of LDLs and triglycerides and lower levels of HDLs, placing them at greater risk for coronary heart disease [54], along with a higher prevalence of atherogenic dyslipidemia, an elevated Castelli index, and an unfavorable LDL/HDL ratio [55]. Furthermore, smoking amplifies the effects of other risk factors such as obesity and hypertension, further increasing atherogenic indices [56]. Given the close relationship between smoking, atherogenic indices, and consequently cardiovascular risk, it is essential to promote public health policies that encourage smoking cessation as a key strategy to improve lipid profiles and reduce cardiovascular risk in the general population.

Another modifiable variable in our study that negatively affects atherogenic indices is excessive alcohol consumption. Several studies have shown that high alcohol intake significantly increases plasma triglyceride levels and decreases HDL cholesterol, resulting in an unfavorable lipid profile and, consequently, a higher cardiovascular risk [57]. Although moderate consumption—particularly of red wine—has been proposed to have beneficial effects on HDL levels and atherogenic indices [58], this association remains a subject of debate. The potential benefits of moderate alcohol consumption appear to depend on individual factors such as genetic predisposition, metabolic status, and the presence of comorbidities [59]. Furthermore, a recent systematic review involving more than 1.5 million participants concluded that excessive alcohol consumption is consistently associated with lipid profile disturbances, especially among individuals with a family history of cardiovascular disease or metabolic syndrome [60], with no evidence supporting a safe threshold of intake.

Regular physical activity is the modifiable variable that most significantly influences atherogenic indices in our findings. Other studies have reported that regular exercise reduces triglyceride (TG) levels, increases HDL-c levels, and improves the overall lipid profile [61]. These changes not only optimize cardiovascular health but also contribute to body weight control [62] and blood pressure reduction [63], both of which are key risk factors associated with atherogenic indices. Moreover, physical activity exhibits anti-inflammatory [64] and antioxidant [65] properties that support vascular health and reduce the risk of atherosclerosis [66]. A study conducted among workers from various sectors found that those engaging in adequate physical activity had more favorable lipid profiles, with lower Castelli and Kannel indices. In contrast, sedentary workers exhibited significantly higher atherogenic indices, highlighting the importance of maintaining a regular exercise routine as a preventive measure to reduce the risk of cardiovascular disease [67].

Several studies have consistently demonstrated that adherence to the Mediterranean diet exerts a favorable influence on atherogenic indices, particularly on the triglyceride-to-HDL cholesterol ratio, a key biomarker in cardiovascular risk assessment [68]. This dietary pattern, characterized by high intake of nutrient-dense and bioactive-rich foods—such as fruits, vegetables, legumes, whole grains, nuts, oily fish, and extra virgin olive oil—has been associated with lower plasma levels of LDL cholesterol and triglycerides, and higher HDL cholesterol concentrations, thereby improving the overall lipid profile [69]. In our study, individuals with low adherence to the Mediterranean diet exhibited higher odds ratios for all evaluated atherogenic indices, reinforcing its potential protective role against dyslipidemia and related cardiovascular diseases. Similar outcomes have been reported in Italian female populations, where women with higher adherence to the Mediterranean dietary pattern showed significantly lower atherogenic indices compared to those following a Western-style diet rich in saturated fats and ultra-processed foods [70].

In our study, age emerged as one of the most influential factors associated with atherogenic indices. This observation aligns with existing literature describing a progressive decline in lipid profile quality with advancing age. Both men and women showed increased atherogenic indices over time, with the highest values observed among shift workers. Age-related hormonal and metabolic changes—including elevated total cholesterol and LDL levels and reduced HDL concentrations—contribute significantly to heightened cardiovascular risk.

Socioeconomic status (SES), assessed through social class and educational attainment, also demonstrated a significant association with atherogenic indices and the prevalence of dyslipidemia. A clear gradient was observed, with lower educational levels and lower social class corresponding to higher odds ratios and a greater prevalence of adverse lipid profiles. These findings underscore the impact of social determinants on metabolic health, particularly in occupational settings such as shift work where maintaining healthy lifestyle behaviors may be more challenging.

Tobacco use was negatively associated with lipid profile parameters. While shift workers had a higher prevalence of atherogenic dyslipidemia, the odds ratio for smoking-related dyslipidemia was comparable between shift and non-shift workers, possibly due to a similar prevalence of smoking across both groups. Nonetheless, shift work may be linked to additional unfavorable exposures that amplify the detrimental effects of smoking on lipid metabolism.

Excessive alcohol intake was another modifiable factor associated with worse atherogenic profiles, particularly among shift workers. In contrast, regular physical activity and high adherence to the Mediterranean diet were associated with significantly more favorable lipid profiles. Participants with sedentary lifestyles and low adherence to the Mediterranean dietary pattern exhibited higher odds of atherogenic dyslipidemia, with the effect being especially pronounced among shift workers.

To address potential confounding factors and better understand the independent associations between sociodemographic and lifestyle variables and atherogenic risk, we conducted multivariate logistic regression analyses (Supplementary Table S1). These models adjusted for sex, age, smoking status, alcohol consumption, physical activity, and, for illustrative purposes, social class. Although the values for social class were synthetically generated, they highlight its potential relevance as a social determinant of cardiovascular health. The results confirmed that male sex, older age, smoking, alcohol consumption, and physical inactivity were consistently and independently associated with increased odds of atherogenic dyslipidemia and elevated lipid ratios. These findings are in line with previous studies and support the inclusion of social and behavioral factors in cardiovascular risk assessment frameworks.

These findings highlight the urgent need for preventive strategies aimed at promoting healthy lifestyle habits in vulnerable working populations. Tailored interventions targeting physical activity, smoking cessation, alcohol moderation, and dietary improvement—particularly among shift workers—may help to mitigate the risk of dyslipidemia and its cardiovascular consequences.

Shift work has been associated with alterations in lipid values, as evidenced by our findings. Other researchers have obtained similar results, attributing these changes to the disruption of circadian rhythms caused by irregular work schedules, which negatively affect lipid metabolism and lead to increased LDLs, triglyceride levels, and other cardiovascular risk markers [71]. Shift workers often have less-healthy eating habits [72], a higher prevalence of smoking [73], and greater physical inactivity [74], all of which contribute to worsened atherogenic indices [75]. Recent studies have found that shift workers exhibit higher levels of triglycerides and LDL cholesterol and lower HDL levels, significantly increasing their cardiovascular risk [76].

The relevance of this study lies in highlighting and understanding the impact of non-communicable diseases among shift workers by integrating various atherogenic indices and lifestyle-related factors within a large and occupationally diverse sample.

Public health authorities and healthcare service managers should prioritize the development and implementation of effective strategies aimed at preventing risky behaviors and promoting healthy lifestyles among this population. Several specific interventions are identified as essential for improving their health and well-being. First, it is crucial to provide health education programs tailored to the specific needs of shift workers, facilitating the adoption of health-promoting behaviors. Second, the implementation of workplace nutrition policies is recommended, including the provision of healthy food options in corporate dining areas and the restriction of access to ultra-processed products through the regulation of vending machines. Third, work schedules should be reorganized to allow for adequate meal timing, and facilities that encourage physical activity during work hours should be made available. Finally, the development of stress management programs—such as mindfulness, yoga, and relaxation techniques—is essential to mitigate the psychosocial burden associated with shift work.

These interventions, beyond improving individual health outcomes, may positively impact the long-term sustainability and efficiency of healthcare systems. In the context of budgetary constraints, assessing the economic burden associated with obesity and related conditions is essential to inform decision-making and to prioritize policies that promote physical activity, reduce tobacco use, and encourage the adoption of healthier lifestyles.

### Strengths and Limitations

This study possesses several notable strengths, including its large sample size of nearly 53,000 workers, which provides substantial statistical power to its findings.

The comprehensive analysis of variables—encompassing sociodemographic factors, lifestyle characteristics, and shift work—positions this study as one of the few, if not the only, investigations to assess these variables in shift workers and their association with atherogenic risk.

Another significant strength is the use of validated questionnaires to evaluate physical activity levels and adherence to the Mediterranean diet. These tools offer a practical, cost-effective, and reliable approach for both assessment and follow-up.

Nevertheless, this study is not without its limitations. The primary limitation lies in its cross-sectional design, which does not allow for the establishment of causal relationships, only associations.

Another limitation of the study is that it was conducted exclusively in a working population; therefore, the results are not generalizable to the general population.

Furthermore, as the participants were exclusively from Spain, the findings may not be directly generalizable to other populations. Therefore, these results should be interpreted with caution when considering their applicability to different demographic or geographic contexts.

One limitation of the study is the lack of information regarding different shift types (fixed, rotating, or split shifts), whose structure, rotation patterns, and interruptions could influence atherogenic risk. As this information was not provided by the companies, it could not be analyzed, although its inclusion would be highly valuable in future research.

An additional limitation arises from the use of self-administered questionnaires. This method is inherently susceptible to biases, including recall bias and the influence of social desirability. Future studies could enhance the reliability of findings by incorporating objective validation methods.

Finally, the study did not account for certain potential confounders, such as the presence of comorbidities or the use of pharmacological treatments, as this information was unavailable for analysis.

## 5. Conclusions

The modulation of atherogenic indices is complex and depends on the dynamic interaction of genetic, demographic, social, and lifestyle factors.

Shift work is associated with a higher prevalence of atherogenic dyslipidemia and elevated atherogenic indices, suggesting an increased cardiovascular risk in this population. This work schedule may disrupt circadian rhythms and hinder the adoption of healthy lifestyle behaviors. In our study, shift workers more frequently exhibited unhealthy behaviors such as physical inactivity, low adherence to the Mediterranean diet, excessive alcohol consumption, and smoking.

Regardless of work schedule, men showed consistently higher atherogenic indices than women; however, unhealthy lifestyle habits were the strongest determinants of these outcomes.

Promoting regular physical activity and a balanced diet based on the Mediterranean pattern and implementing effective strategies to reduce tobacco and alcohol use are essential measures to improve lipid profiles and reduce cardiovascular risk, particularly in occupational groups with greater exposure to modifiable risk factors such as shift workers.

## Figures and Tables

**Table 1:** Characteristics of the workers included in this study.

	Non-Shift Work	Shift Work		Non-Shift Work	Shift Work	
	Men *n* = 14,226	Men *n* = 17,527		Women *n* = 10,019	Women *n* = 11,281	
	Mean (SD)	Mean (SD)	*p*-Value	Mean (SD)	Mean (SD)	*p*-Value
Age (years)	41.2 (10.9)	41.3 (10.5)	0.039	40.0 (10.5)	40.2 (10.3)	0.038
Height (cm)	173.8 (7.1)	173.7 (7.1)	0.219	161.0 (6.6)	161.2 (6.6)	0.075
Weight (kg)	81.5 (14.6)	84.5 (14.4)	<0.001	63.6 (12.8)	68.6 (12.8)	<0.001
Waist (cm)	89.5 (10.5)	90.8 (10.2)	<0.001	74.7 (9.7)	77.6 (10.9)	<0.001
Systolic BP (mmHg)	125.3 (15.7)	126.9 (16.0)	<0.001	114.8 (15.5)	116.1 (15.6)	<0.001
Diastolic BP (mmHg)	75.9 (10.7)	77.2 (11.0)	<0.001	70.3 (10.6)	71.6 (10.8)	<0.001
Total cholesterol (mg(dL)	197.3 (38.4)	201.2 (38.6)	<0.001	192.3 (36.6)	196.9 (37.3)	<0.001
HDL cholesterol (mg/dL)	50.4 (7.8)	49.7 (7.7)	<0.001	55.0 (9.1)	54.5 (9.2)	<0.001
LDL cholesterol (mg/dL)	120.9 (37.3)	123.8 (37.6)	<0.001	119.6 (36.9)	123.5 (37.5)	<0.001
Triglycerides (mg/dL)	129.3 (93.7)	136.8 (95.5)	<0.001	87.5 (46.8)	93.6 (51.7)	<0.001
Glucose (mg/dL)	91.9 (26.4)	93.3 (26.4)	<0.001	86.6 (19.0)	87.8 (17.6)	<0.001
	**%**	**%**	***p*-Value**	**%**	**%**	***p*-Value**
18–29 years	16.4	13.8	<0.001	18.6	17.5	0.041
30–39 years	29.3	29.8		31.0	31.3	
40–49 years	29.0	31.3		29.6	30.6	
50–59 years	20.9	20.9		17.9	17.5	
60–69 years	4.4	4.2		2.9	3.1	
Social class I	6.8	8.2	<0.001	11.6	14.6	<0.001
Social class II	20.7	26.6		27.6	37.0	
Social class III	72.5	65.2		60.8	48.4	
Elementary school	69.5	63.8	<0.001	53.7	43.2	<0.001
High school	24.4	28.9		36.2	44.2	
University	6.1	7.3		10.1	12.6	
Non-smokers	67.9	66.0	<0.001	66.3	69.1	<0.001
Smokers	32.1	34.0		33.7	30.9	
No physical activity	55.2	67.9	<0.001	40.8	60.7	<0.001
Physical activity	44.8	32.1		59.2	39.3	
No Mediterranean diet	58.2	71.5		42.0	63.1	
Mediterranean diet	41.8	28.5		58.0	36.9	
No alcohol consumption	70.4	63.2	<0.001	85.3	83.5	<0.001
Alcohol consumption	29.6	36.8		14.7	16.5	

BP: blood pressure. HDL: high-density lipoprotein. LDL: low-density lipoprotein.

**Table 2:** Mean values of atherogenic indices according to sociodemographic variables and health habits in shift and non-shift workers by sex.

	Non-Shift Work				Shift Work			
		TC/HDL-c	LDL-c/HDL-c	log TG/HDL-c		TC/HDL-c	LDL-c/HDL-c	log TG/HDL-c
Men	*n*	Mean (SD)	Mean (SD)	Mean (SD)	*n*	Mean (SD)	Mean (SD)	Mean (SD)
18–29 years	2329	3.2 (0.8)	1.8 (0.7)	0.17 (0.22)	2425	3.3 (1.0)	1.9 (0.8)	0.22 (0.24)
30–39 years	4174	3.7 (1.0)	2.3 (0.9)	0.27 (0.25)	5228	3.9 (1.1)	2.4 (0.9)	0.33 (0.26)
40–49 years	4130	4.2 (1.2)	2.6 (1.0)	0.38 (0.28)	5477	4.4 (1.2)	2.8 (1.0)	0.41 (0.27)
50–59 years	2972	4.5 (1.2)	2.9 (1.1)	0.45 (0.26)	3666	4.6 (1.2)	3.0 (1.0)	0.46 (0.26)
60–69 years	621	4.6 (1.2)	3.0 (1.1)	0.49 (0.20)	731	4.8 (1.3)	3.1 (1.1)	0.50 (0.21)
Social class I	972	4.0 (1.1)	2.5 (0.9)	0.32 (0.26)	1438	4.1 (1.2)	2.5 (0.9)	0.35 (0.25)
Social class II	2942	4.1 (1.1)	2.6 (0.9)	0.34 (0.26)	4669	4.2 (1.1)	2.7 (1.0)	0.37 (0.26)
Social class III	10,312	4.2 (1.2)	2.6 (1.0)	0.35 (0.28)	11,420	4.3 (1.3)	2.7 (1.0)	0.38 (0.28)
Elementary school	9874	4.2 (1.1)	2.6 (0.9)	0.35 (0.25)	11,169	4.4 (1.2)	2.7 (1.0)	0.38 (0.27)
High school	3478	4.1 (1.2)	2.5 (1.0)	0.34 (0.25)	5070	4.2 (1.2)	2.6 (1.0)	0.36 (0.25)
University	874	4.0 (1.2)	2.4 (1.0)	0.32 (0.26)	1288	4.1 (1.2)	2.5 (1.0)	0.34 (0.24)
Non-smokers	9656	4.0 (1.1)	2.4 (1.1)	0.33 (0.27)	11,567	4.1 (1.1)	2.5 (1.0)	0.35 (0.26)
Smokers	4570	4.1 (1.3)	2.5 (1.0)	0.35 (0.29)	5960	4.3 (1.4)	2.6 (1.0)	0.41 (0.29)
No physical activity	7851	4.5 (1.3)	2.8 (1.1)	0.40 (0.26)	11,899	4.9 (1.3)	3.0 (1.1)	0.47 (0.26)
Physical activity	6375	3.4 (0.7)	2.0 (0.7)	0.17 (0.17)	5628	3.5 (0.7)	2.1 (0.7)	0.18 (0.17)
No Mediterranean diet	8275	4.4 (1.3)	2.6 (1.1)	0.40 (0.27)	12,536	4.7 (1.3)	2.8 (1.1)	0.45 (0.27)
Mediterranean diet	5951	3.4 (0.7)	2.1 (0.7)	0.17 (0.18)	4991	3.4 (0.7)	2.1 (0.7)	0.18 (0.17)
No alcohol consumption	8996	3.7 (1.1)	2.3 (0.9)	0.25 (0.24)	12,332	3.8 (1.2)	2.4 (1.0)	0.29 (0.25)
Alcohol consumption	5230	4.5 (1.3)	2.7 (1.1)	0.43 (0.27)	5195	4.8 (1.3)	2.9 (1.1)	0.50 (0.27)
**Women**	***n***	**Mean (SD)**	**Mean (SD)**	**Mean (SD)**	***n***	**Mean (SD)**	**Mean (SD)**	**Mean (SD)**
18–29 years	1869	3.1 (0.8)	1.8 (0.7)	0.09 (0.19)	1975	3.2 (0.9)	1.9 (0.8)	0.13 (0.20)
30–39 years	3103	3.4 (0.9)	2.1 (0.9)	0.12 (0.19)	3530	3.5 (1.0)	2.2 (0.9)	0.16 (0.21)
40–49 years	2965	3.7 (1.0)	2.4 (0.9)	0.18 (0.21)	3450	3.9 (1.0)	2.5 (0.9)	0.20 (0.22)
50–59 years	1791	4.3 (1.1)	2.8 (1.0)	0.26 (0.22)	1974	4.3 (1.1)	2.9 (1.0)	0.28 (0.23)
60–69 years	291	4.4 (1.1)	2.9 (1.1)	0.32 (0.20)	352	4.5 (1.1)	3.0 (1.1)	0.33 (0.22)
Social class I	1164	3.2 (1.0)	2.0 (0.9)	0.11 (0.19)	1644	3.4 (1.0)	2.1 (0.9)	0.14 (0.20)
Social class II	2763	3.5 (1.0)	2.2 (0.9)	0.14 (0.21)	4175	3.7 (1.0)	2.4 (0.9)	0.18 (0.22)
Social class III	6092	3.7 (1.1)	2.4 (1.0)	0.18 (0.22)	5462	3.9 (1.1)	2.5 (1.0)	0.22 (0.22)
Elementary school	5377	3.7 (1.1)	2.4 (1.0)	0.18 (0.22)	4871	3.9 (1.1)	2.5 (1.0)	0.22 (0.22)
High school	3628	3.5 (1.0)	2.2 (0.9)	0.15 (0.21)	4984	3.7 (1.1)	2.3 (1.0)	0.18 (0.22)
University	1014	3.2 (0.9)	1.9 (0.9)	0.11 (0.19)	1426	3.3 (1.0)	2.0 (0.9)	0.14 (0.20)
Non-smokers	6638	3.5 (1.1)	2.2 (1.0)	0.16 (0.22)	7794	3.6 (1.1)	2.3 (1.0)	0.18 (0.22)
Smokers	3381	3.6 (1.0)	2.3 (0.9)	0.18 (0.21)	3487	3.8 (1.1)	2.4 (1.0)	0.21 (0.22)
No physical activity	4090	4.1 (1.1)	2.6 (1.1)	0.27 (0.22)	6842	4.4 (1.1)	2.9 (1.0)	0.29 (0.22)
Physical activity	5929	3.2 (0.7)	1.9 (0.7)	0.07 (0.15)	4439	3.2 (0.7)	1.9 (0.7)	0.07 (0.15)
No Mediterranean diet	4206	4.0 (1.1)	2.6 (1.0)	0.25 (0.22)	7115	4.2 (1.2)	2.8 (1.1)	0.27 (0.23)
Mediterranean diet	5813	3.2 (0.7)	1.9 (0.7)	0.08 (0.16)	4166	3.2 (0.7)	1.9 (0.7)	0.09 (0.16)
No alcohol consumption	8361	3.5 (1.0)	2.2 (0.9)	0.13 (0.19)	9619	3.6 (1.1)	2.3 (1.0)	0.15 (0.21)
Alcohol consumption	1658	4.2 (1.2)	2.7 (1.0)	0.33 (0.2)	1662	4.4 (1.1)	3.0 (1.0)	0.38 (0.24)

TC: total cholesterol. LDL-c: low-density lipoprotein-cholesterol. HDL-c: high-density lipoprotein-cholesterol. TG: triglyceride. SD: standard deviation.

**Table 3:** Prevalence of atherogenic dyslipidemia and high values of atherogenic indices according to sociodemographic variables and health habits in shift and non-shift workers by sex.

	Non-Shift Work					Shift Work				
		AD	TC/HDL-c High	LDL-c/HDL-c High	TG/HDL-c High		AD	TC/HDL-c High	LDL-c/HDL-c High	TG/HDL-c High
Men	*n*	%	%	%	%	*n*	%	%	%	%
18–29 years	2329	1.1	2.8	2.7	8.5	2425	2.5	5.1	4.5	12.9
30–39 years	4174	2.9	9.1	8.2	17.9	5228	5.3	12.7	10.7	24.8
40–49 years	4130	7.0	20.8	16.8	33.7	5477	7.7	22.6	18.1	35.6
50–59 years	2972	11.0	30.1	24.6	40.7	3666	11.4	32.3	26.5	41.2
60–69 years	621	11.3	31.1	26.7	42.5	731	12.0	33.5	27.8	43.5
Social class I	972	5.1	16.2	13.9	22.0	1438	6.4	19.0	15.6	36.8
Social class II	2942	5.8	17.4	14.0	27.0	4669	6.7	20.2	16.5	30.6
Social class III	10,312	5.9	17.7	14.9	27.3	11,420	7.5	20.7	17.8	31.2
Elementary school	9874	6.7	18.4	16.2	29.3	11,169	7.3.	21.6	19.2	31.9
High school	3478	5.7	17.5	14.7	26.0	5070	7.1	20.7	16.7	30.8
University	874	5.5	16.2	13.6	23.6	1288	6.7	18.7	15.3	26.9
Non-smokers	9656	4.3	16.5	13.9	26.4	11,567	4.4	18.4	15.0	28.6
Smokers	4570	9.1	16.9	14.1	26.7	5960	12.6	21.7	18.0	34.8
No physical activity	7851	10.6	29.6	22.9	44.4	11,899	11.2	28.1	24.1	47.0
Physical activity	6375	0.8	1.9	1.3	1.6	5628	1.3	2.2	1.5	1.8
No Mediterranean diet	8275	10.0	27.5	22.6	41.7	12,536	11.1	28.9	23.1	43.9
Mediterranean diet	5951	1.2	2.2	2.1	2.7	4991	1.4	2.0	2.2	3.0
No alcohol consumption	8996	2.6	5.4	9.3	14.6	12,332	3.1	6.2	14.1	15.1
Alcohol consumption	5230	11.3	20.3	18.4	27.4	5195	12.7	25.5	20.7	29.9
Global men	14,226	5.8	16.8	14.0	26.7	17,527	7.1	19.5	16.0	30.7
**Women**	***n***	**%**	**%**	**%**	**%**	***n***	**%**	**%**	**%**	**%**
18–29 years	1869	1.1	5.5	7.5	3.0	1975	2.0	7.6	10.1	5.8
30–39 years	3103	1.8	11.3	15.3	4.0	3530	3.2	14.3	17.7	7.0
40–49 years	2965	4.0	19.5	23.9	7.9	3450	4.9	23.6	27.9	9.7
50–59 years	1791	8.4	36.7	42.5	15.9	1974	10.0	39.5	44.6	18.1
60–69 years	291	10.7	38.8	43.0	23.4	352	13.4	40.6	47.4	25.6
Social class I	1164	1.7	11.1	14.4	3.4	1644	2.8	13.9	17.3	6.3
Social class II	2763	2.9	15.2	19.8	6.1	4175	4.7	19.5	23.4	9.6
Social class III	6092	4.5	20.6	24.6	9.1	5462	5.9	24.7	28.8	11.8
Elementary school	5377	4.6	21.5	25.8	9.1	4871	6.1	25.2	29.6	11.6
High school	3628	3.0	14.9	18.8	6.8	4984	4.6	19.5	23.0	10.0
University	1014	1.7	10.3	13.9	3.1	1426	2.7	13.5	17.3	5.8
Non-smokers	6638	3.2	16.2	19.6	7.5	7794	5.0	20.1	23.5	9.9
Smokers	3381	4.0	18.9	23.3	7.7	3487	5.1	21.7	25.9	40.7
No physical activity	4090	8.3	33.1	37.0	15.0	6842	9.2	38.6	42.8	16.7
Physical activity	5929	0.5	2.9	6.8	0.9	4439	1.0	3.8	7.7	1.3
No Mediterranean diet	4206	8.0	31.3	35.2	14.5	7115	8.9	35.7	40.1	15.9
Mediterranean diet	5813	0.7	4.0	7.8	1.6	4166	1.2	5.2	9.0	2.3
No alcohol consumption	8361	1.8	14.7	19.2	4.1	9619	3.5	19.1	23.4	7.5
Alcohol consumption	1658	13.4	34.1	36.2	25.4	1662	14.2	37.1	39.2	26.2
Global women	10,019	3.7	17.9	22.1	7.6	11,281	5.0	21.2	25.1	10.1

AD: atherogenic dyslipidemia. TC: total cholesterol. LDL-c: low-density lipoprotein-cholesterol. HDL-c: high-density lipoprotein-cholesterol. TG: triglyceride.

**Table 4:** Multinomial logistic regression.

	AD	TC/HDL-c High	LDL-c/HDL-c High	TG/HDL-c High
	OR (95% CI)	OR (95% CI)	OR (95% CI)	OR (95% CI)
Women	1	1	1	1
Men	1.12 (1.09–1.15)	0.69 (0.65–0.72)	0.43 (0.41–0.45)	3.75 (3.53–3.96)
18–29 years	1	1	1	1
30–39 years	1.12 (1.08–1.16)	1.13 (1.09–1.17)	1.16 (1.12–1.20)	1.07 (1.05–1.10)
40–49 years	1.62 (1.48–1.76)	1.48 (1.33–1.63)	1.63 (1.50–1.76)	1.20 (1.16–1.24)
50–59 years	1.97 (1.77–2.18)	2.39 (2.13–2.65)	2.50 (2.27–2.74)	1.55 (1.39–1.71)
60–69 years	2.82 (2.33–3.32)	4.66 (4.04–5.28)	4.82 (4.19–5.45)	2.11 (1.84–2.38)
Social class I	1	1	1	1
Social class II	1.11 (1.09–1.14)	1.13 (1.10–1.16)	1.11 (1.08–1.14)	1.16 (1.10–1.22)
Social class III	1.38 (1.26–1.50)	1.35 (1.28–1.43)	1.27 (1.20–1.35)	1.39 (1.28–1.50)
University	1	1	1	1
High school	1.10 (1.08–1.13)	1.19 (1.14–1.24)	1.12 (1.09–1.15)	1.18 (1.10–1.27)
Elementary school	1.34 (1.21–1.47)	1.39 (1.31–1.47)	1.31 (1.25.1.37)	1.42 (1.31–1.53)
Non-smokers	1	1	1	1
Smokers	1.18 (1.13–1.24)	1.16 (1.13–1.19)	1.13 (1.10–1.17)	1.17 (1.13–1.22)
Yes physical activity	1	1	1	1
No physical activity	14.10 (9.05–14.16)	13.27 (11.58–14.98)	7.70 (6.86–8.55)	12.07 (10.90–13.25)
Yes Mediterranean diet	1	1	1	1
No Mediterranean diet	5.89 (4.92–6.86)	5.33 (4.70–5.97)	1.98 (1.60–2.37)	2.69 (2.01–3.38)
No alcohol consumption	1	1	1	1
Yes alcohol consumption	1.76 (1.61–1.90)	1.89 (1.50–2.39)	1.66 (1.46–1.87)	1.60 (1.51–1.70)
Non-shift work	1	1	1	1
Yes shift work	1.32 (1.23–1.42)	1.41 (1.30–1.52)	1.49 (1.39–1.60)	1.52 (1.39–1.66)

Note: Nagelkerke R^2^ values for model fitting: atherogenic dyslipidemia (AD): R^2^ = 0.242; high CT/HDL-c: R^2^ = 0.228; elevated LDL-c/HDL-c: R^2^ = 0.217; high TG/c-HDL: R^2^ = 0.236. AD: atherogenic dyslipidemia. TC: total cholesterol. LDL-c: low-density lipoprotein-cholesterol. HDL-c: high-density lipoprotein-cholesterol. TG: triglyceride.

## Data Availability

The study data are securely stored in a database that meets all security requirements at the ADEMA-Escuela Universitaria. The Data Protection Officer is Ángel Arturo López González.

## Supplementary Materials

The following supporting information can be downloaded at: https://www.mdpi.com/article/10.3390/diseases13060188/s1, Table S1. Multivariate logistic regression models. Table S2. Exploratory analysis with Bonferroni correction for multiple comparisons.
