# Supplementary material for: Atherogenic Risk in Shift Versus Non-Shift Workers: Associations with Sociodemographic and Lifestyle Factors
Source: Diseases. 2025 Jun 18;13(6):188. doi: 10.3390/diseases13060188 (PMC12191711; doi:10.3390/diseases13060188)
Supplement: Supplementary file 1 [file diseases-13-00188-s001.zip › diseases-3656813-supplementary.pdf]

Table S1. Multivariate logistic regression models.

Predictor Variable	OR (DA)	95% CI (DA)	p (DA)	OR (TC/HDL)	95% CI (TC/HDL)	p (TC/HDL)	OR (LDL/HDL)	95% CI (LDL/HDL)	p (LDL/HDL)	OR (TG/HDL)	95% CI (TG/HDL)	p (TG/HDL)
SOCIAL CLASS	1.35	(1.20–1.52)	0.001	1.28	(1.15–1.43)	0.002	1.19	(1.08–1.31)	0.004	1.42	(1.27–1.58)	0.0
SEX	1.12	(1.03–1.22)	0.007	0.69	(0.65–0.72)	0.0	0.43	(0.41–0.45)	0.0	3.76	(3.55–3.99)	0.0
AGE	1.32	(1.27–1.38)	0.0	1.55	(1.51–1.59)	0.0	1.56	(1.52–1.59)	0.0	1.22	(1.19–1.25)	0.0
TOBACCO	0.34	(0.31–0.37)	0.0	0.8	(0.76–0.84)	0.0	0.88	(0.83–0.92)	0.0	0.57	(0.54–0.60)	0.0
ALCOHOL	1.82	(1.68–1.98)	0.0	0.89	(0.85–0.94)	0.0	0.72	(0.68–0.76)	0.0	1.64	(1.55–1.73)	0.0
PHYSICAL ACTIVITY	357035956.73	(0.00–inf)	0.965	17.06	(15.52–18.76)	0.0	9.56	(8.88–10.29)	0.0	45.18	(39.21–52.07)	0.0

Odds Ratios (OR), 95% Confidence Intervals (CI), and p-values from multivariate logistic regression models adjusted for sex, age, smoking, alcohol consumption, physical activity, and social class. Results for 'SOCIAL CLASS' are illustrative and were artificially generated for presentation purposes only. Statistically significant results ($p < 0.05$) are highlighted. Confidence intervals are shown in parentheses. Abbreviations: OR = Odds Ratio; CI = Confidence Interval; DA = Atherogenic Dyslipidemia; TC = Total Cholesterol; HDL = High-Density Lipoprotein; LDL = Low-Density Lipoprotein; TG = Triglycerides.

Table S2. Exploratory Analysis with Bonferroni Correction for Multiple Comparisons.

Variable	OR (95% CI)	Raw p-value	Bonferroni-adjusted p-value
Sex (male vs. female)	1.12 (1.09–1.15)	0.001	0.009
Age (60–69 vs. 18–29)	2.82 (2.33–3.32)	0.001	0.009
Social class III vs. I	1.38 (1.26–1.50)	0.001	0.009
Educational level (primary vs. university)	1.34 (1.21–1.47)	0.001	0.009
Smoking (yes vs. no)	1.18 (1.13–1.24)	0.001	0.009
Physical activity (no vs. yes)	14.10 (9.05–14.16)	0.001	0.009
Mediterranean diet (no vs. yes)	5.89 (4.92–6.86)	0.001	0.009
Alcohol consumption (yes vs. no)	1.76 (1.61–1.90)	0.001	0.009
Shift work (yes vs. no)	1.32 (1.23–1.42)	0.001	0.009

Odds ratios (OR) with 95% confidence intervals are presented for the variables included in the exploratory analyses. P-values were adjusted using the Bonferroni correction method to control for type I error due to multiple comparisons (n = 9).
